# Achieving Good Practice in Legislation, Law Enforcement and Driving Behaviour Interventions

**Published:** 2019-08

**Authors:** Ray Shuey

**Affiliations:** ^*a*^Principal and Founder, Strategic Safety Solutions Pty Ltd. Australia.

## Abstract

Achieving good practice in road safety reform requires a holistic approach, strong partnerships, integrated systems with effective enforcement and education programs. The objective is to influence road user behaviours so as to adopt a culture of safety and an attitude of respect. Respect for self, respect for passengers and importantly, respect for other road users, ensuring safety for all.

The International “Safe Systems Approach” provides guidance and direction in improving the road network, vehicle design and road user behaviours. This presentation will provide focus on road user behaviours and particularly high-risk activities.

Reform must be founded on a dynamic and active national road safety council endorsed with the political will of government and vested with the power base to direct road safety authorities, a funding base to ensure achievement and all with the common purpose of “saving lives” and towards “Vision Zero”. Altruistic aims must convert to practical and actionable items with realistic performance indicators for all partner agencies.

Road safety research withstanding the test of time supports the “deterrence” theory in the practical application through education and enforcement strategies ensuring that drivers are compliant with the legislation and deterred from committing high-risk behavioural offences which jeopardise the lives of themselves and others.

To achieve this outcome in a conceptual framework, you must increase the perception ‘anywhere, anytime and for anybody’. If you commit a high-risk offence you will be caught and punished. This perception of being caught and sanctioned is a primary focus in achieving compliance until the community adopts a focus of self-regulation i.e. you wear your seat-belt because of safety, not because of any penalty; you do not speed because it is safer for yourself and others not because of any perceived penalty. This is the end result of impacting on human behaviours in a safe road user environment.

This process requires sound, practical and implementable laws to ensure guidance and an effective penalty base. It must be supported by education and enforcement strategies operating to ensure road users understand the rationale for safety restrictions, with enforcement that is swift, effective and meaningful. The enforcement aim is to change road user behaviours to ensure a safety environment.

The principles of effective enforcement are: 1. Highly visible and active enforcement (drivers must see visible enforcement); 2. Repeated often (frequency is mandatory); 3. Be fair and consistent (No bargaining, no bribery, no corruption and no special treatments) and; 4. Well publicised (using media and social media to inform, educate and provide awareness). All elements are essential for successful road safety reform. Several good practice interventions will be discussed demonstrating the beneficial outcomes of these strategies.

**Keywords:**

Safe Systems Approach, Good Practice, Legislation, Law Enforcement, Driving Behaviour

